# A critical interpretive synthesis of power and mistreatment of women in maternity care

**DOI:** 10.1371/journal.pgph.0000616

**Published:** 2023-01-30

**Authors:** Marta Schaaf, Maayan Jaffe, Özge Tunçalp, Lynn Freedman

**Affiliations:** 1 Independent Consultant, Brooklyn, New York, United States of America; 2 Department of Sexual and Reproductive Health and Research, UNDP/UNFPA/UNICEF/WHO/World Bank Special Programme of Research, Development and Research Training in Human Reproduction (HRP), World Health Organization, Geneva, Switzerland; 3 Heilbrunn Department of Population and Family Health, Columbia University Mailman School of Public Health, New York, New York, United States of America; Jhpiego, UNITED STATES

## Abstract

Labouring women may be subjected to physical and verbal abuse that reflects dynamics of power, described as Mistreatment of Women (MoW). This Critical Interpretive Synthesis on power and MoW consolidates current research and advances theory and practice through inter-disciplinary literature exploration. The review was undertaken in 3 phases. Phase 1 consisted of topic scoping; phase 2 entailed exploration of key power-related drivers emerging from the topic scoping; and phase 3 entailed data synthesis and analysis, with a particular focus on interventions. We identified 63 papers for inclusion in Phase 1. These papers utilized a variety of methods and approaches and represented a wide range of geographic regions. The power-related drivers of mistreatment in these articles span multiple levels of the social ecological model, including intrapersonal (e.g. lack of knowledge about one’s rights), interpersonal (e.g. patient-provider hierarchy), community (e.g. widespread discrimination against indigenous women), organizational (e.g. pressure to achieve performance goals), and law/policy (e.g. lack of accountability for rights violations). Most papers addressed more than one level of the social-ecological model, though a significant minority were focused just on interpersonal factors. During Phase 1, we identified priority themes relating to under-explored power-related drivers of MoW for exploration in Phase 2, including lack of conscientization and normalization of MoW; perceptions of fitness for motherhood; geopolitical and ethnopolitical projects related to fertility; and pressure to achieve quantifiable performance goals. We ultimately included 104 papers in Phase 2. The wide-ranging findings from Phase 3 (synthesis and analysis) coalesce in several key meta-themes, each with their own evidence-base for action. Consistent with the notion that research on power can point us to “drivers of the drivers,” the paper includes some intervention-relevant insights for further exploration, including as relating to broader social norms, health systems design, and the utility of multi-level strategies.

## Introduction

Public health actors–ranging from researchers to multilateral institutions—increasingly acknowledge the notion that power matters to public health [1]. While biomedical approaches to evidence generation traditionally focus on proximal, specific determinants of health outcomes, social science researchers and advocates note that distal, amorphous determinants–such as the distribution of power in health policies and systems and in society more broadly—shape health sector priorities; health service coverage, content, quality; and, ultimately, health outcomes [1, 2]. Mistreatment of Women (MoW) in maternity care is a paradigmatic example of how power is expressed in health systems; labouring women may be subjected to hitting, slapping, verbal insults, and other behaviors that explicitly or implicitly reflect and perpetuate the dynamics of power in the labour ward and society more broadly [3].

Power has been defined and assessed in myriad ways in health research. Rather than defining power itself, researchers often examine “sources” or “flows” of power and how they shape the universe of proximal and distal health determinants, such as research on global health governance, commercial power and other social determinants of health, health agenda setting, health policy implementation, and patient-provider interactions [1, 4–8]. There is a deep well of theoretical debates regarding power in the political sciences, but in the context of health research, it can be helpful to think of two main facets of power—*power over* and *power to* [1]. *Power over* is frequently conceptualized in two ways—as a ubiquitous and diffuse force shaping the context in which people live and the choices they can make, as, for example, described by Foucault [8], or, as an interface or conflict among actors, wherein one actor uses power to prevent others from gaining or exercising their own [9–11]. *Power to* is often defined as agency, self or collective efficacy, people power, or empowerment in the exercise of rights and entitlements [11]. Concepts of power can be applied to all levels of the social ecological model of health, encompassing intrapersonal, interpersonal, institution/organization, community, and law/policy dynamics [12, 13]. Power is a neutral concept; power dynamics can be contested, subverted, negotiated and mobilized in ways that undermine or support the rights of women in maternity care.

A significant body of quantitative and qualitative research has explored drivers and behaviors of MoW as manifestations of *power over*, with particular focus on harmful power asymmetries, such as gender and other social hierarchies and punitive management within the health sector hierarchy [13–17]. Some interventions promoting respectful maternity care explicitly seek to alter prevailing power dynamics and enhance *power to*, such as by empowering patients to demand better care; by providing materials or training to empower providers, particularly low status providers; or by using legal or other approaches to exact accountability for mistreatment [18–20]. Much theory and practice relating to *power to* come from the Brazilian educator and philosopher Paulo Freire’s concept of conscientization. Conscientization refers to an understanding of one’s reality in a way that allows one to intervene and change it. More specifically, achieving critical consciousness allows one to take action against oppressive systems and actors [21].

In conjunction with robust research on proximal, specific determinants, research on power dynamics can turn our focus to fundamental causes, suggesting avenues for transformational change [1, 3, 16, 21–23]. Without an examination of power, health systems scholarship, including on MoW, can generate misleading narratives that emphasize factors such as patient attributes, patient mistrust, biological race, and implicit biases as most salient drivers of the quality of care and patient outcomes [24]. While many fundamental causes fall outside of what is traditionally considered the remit of the health system, identifying and exploring such causes is crucial to identifying appropriate policy responses, such as changing health care worker training, supervision, or professional scope of practice; or, health promotion; social policy; and anti-discrimination strategies [2, 14].

This Critical Interpretive Synthesis (CIS) on power and MoW aims to consolidate current research on how power dynamics shape and MoW and how these can be disrupted. This paper is paper is part of a special collection of articles exploring strategies to reduce mistreatment of women during childbirth and to improve respectful care. More specifically, the paper starts by scoping the mixed methods literature on power-related drivers of MoW. It then explores multi-disciplinary bodies of literature addressing key drivers we identify. These findings can be used to inform policy-making as well as further descriptive and evaluation research.

## Methods

CIS is a type of review that integrates methods from review methodology and qualitative research with an aim to critically synthesize, develop analytic propositions, and contribute to theory building; in the context of this paper, we focus on the development of program theory [25–27]. CIS allows for an iterative and dynamic approach to research question development and selection of materials for inclusion in reviews [25, 28]. Preliminary findings are used to inform new areas of research [26]. CIS was selected as the method for this paper as we sought to review a heterogenous set of literature on drivers and approaches to change.

The authors jointly developed the following guiding questions for the review:

- How does the peer-reviewed literature describe key power-related drivers of MoW during pregnancy and childbirth?
- How are the power-related drivers theorized and addressed within and outside of the literature focused explicitly on MoW?
- What lessons can be drawn from this literature to inform strategies to reduce MoW?

**Fig 1:**
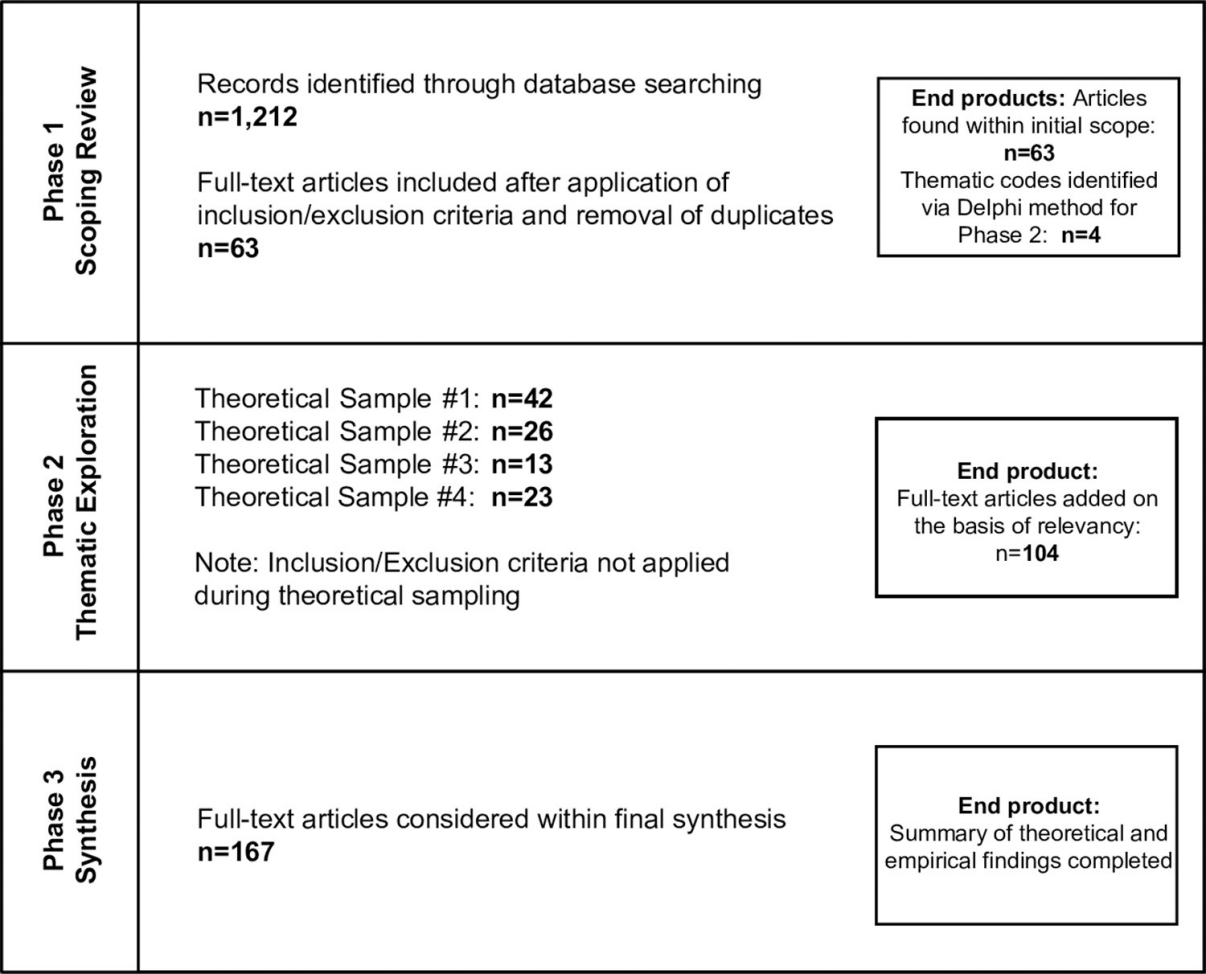
Summary of review phases.

We undertook our review in 3 phases. Phase 1 consisted of topic scoping; phase 2 entailed exploration of key power-related drivers emerging from the topic scoping and review of interventions to address these drivers; and phase 3 entailed data synthesis and analysis, with particular focus on interventions. The protocol for this synthesis is registered on the International Prospective Register of Systematic Reviews (PROSPERO), CRD42021224847. Fig 1 summarizes the three phases of this review and how later phases built on earlier phases.

### Phase 1: Topic scoping

Phase 1 was a mixed methods scoping review that is reproducible based on the methods presented here. As per CIS methodology, the criteria for article inclusion in all three phases were determined by subject matter relevance and the likelihood that the article would contribute to theory development [25].

In Phase 1, the following criteria were applied for inclusion:

- Studies and papers published regarding Low- and Middle-Income Countries (LMICs)
- Studies relating to MoW as described by Bohren et al. [3]
- Studies investigating power dynamics in the context of MoW, irrespective of whether or not the word “power” was used
- Published since 2005 (through November 2020)
- Published in a peer reviewed journal

Since important theorizing and discussions regarding power and MoW have occurred in commentaries and reviews, we did not apply exclusion criteria with regards to the type of paper.

As summarized in Table 1, we developed search terms related to MoW, pregnancy and childbirth, power, and drivers of MoW, and used MESH terms for LMICs. We undertook our search in PubMed, generating 481 initial results; and in Scopus, generating 330 initial results respectively.

**Table 1:** Search terms.

**Mistreatment of Women**	**AND**	**Pregnancy and childbirth**	**AND**	**Power**	**AND**	**MESH terms for LMICs**
“Disrespect and abuse” OR “violence” OR “respectful care” OR “abuse” OR “discrim*” OR “stigma” OR “mistreatment”		"pregnan*" OR "matern*" OR "obstetric" OR “childbirth”		“power” OR "inequalit*" OR "structur* OR “discrimination"		

To minimize individual reviewer bias and to decrease ambiguity about what constitutes “power dynamics,” two reviewers independently assessed a preliminary sample of 20 papers. They then discussed any discrepancies and their rationales for inclusion and exclusion. This helped to ensure consistent application of inclusion criteria.

Sixty papers ultimately met our inclusion criteria and were imported in Zotero. We also ran the search terms in Google Scholar, which uses a different algorithm, to ascertain the completeness of our results. We sorted the 398 results by relevance and stopped reviewing at result 119 after we deemed 50 results in a row to be irrelevant. We included 3 additional papers from Google Scholar. Finally, we reviewed the reference lists of included studies, and did not identify any additional articles to include.

The data from the 63 included papers were recorded in an extraction tool that included fields related to the power-related drivers identified in the article, the theory applied (if any), and lessons and conclusions related to power. After extracting all of the articles, all authors on the paper discussed and identified emergent themes related to the drivers, such as prejudices held about particular social groups, and lack of accountability structures.

### Phase 2: Thematic exploration using theoretical sampling

We utilized a modified Delphi process to refine the themes emerging from the Phase 1 scoping review. To do so, we shared the list of themes that arose from the Phase 1 articles with experts in the field of MoW, namely the WHO Working Group on Interventions to Reduce Mistreatment of Women During Childbirth. In particular, we sought to prioritize themes that the Working Group felt are comparatively under-researched in the MoW literature, including avoiding themes that were going to be otherwise deeply explored in this Special Collection. For example, themes that emerged during Phase 1 that we determined to be comparatively well-researched included stress and burnout among providers. We favored themes that might be particularly pertinent to the development of future hypotheses, research, programs, and policy addressing MoW. Example themes that we decided were relatively less pertinent included linguistic differences between patient and provider, i.e. situations where the patient and provider did not have a language in common. The themes we selected were not mutually exclusive. Following discussion with the group, we collapsed some themes into groups. For example, we collapsed lack of patient conscientization regarding MoW and patient and provider normalization of MoW together. Though these themes are somewhat conceptually distinct, they are often described as related (e.g. patient normalization of MoW stems from patient lack of conscientization) and they might be addressed by similar strategies.

Following the Delphi process, we reached consensus around four key inter-related themes that the group felt were most theoretically and programmatically useful to explore further: (1) lack of conscientization regarding MoW and normalization of MoW, (2) perceptions regarding fitness for motherhood, (3) geopolitical and ethnopolitical projects related to fertility, (4) pressure to achieve quantifiable performance goals.

We then created a new set of search strategies for each of these themes, and used a theoretical sampling approach to situate the power-related drivers of MoW in a larger social, institutional, and conceptual context [29–31]. These “mini reviews” relied on PubMed, GoogleScholar, Lexis-Nexis and citation chaining to explore a multi-disciplinary body of literature including global health, law, and organizational behavior journals. We started by searching the terms included in the name of the themes and then–using citation chaining or the addition of the words “interventions,” “program,” and “policy,” followed threads that emerged, particularly those related to the evidence base for policies and programs that have been employed to address the power-related driver. The original inclusion and exclusion criteria applied to Phase 1 were deliberately not applied to Phase 2, in part because the authors sought to include evidence beyond of the traditional maternal health literature and did not want to limit any potentially relevant results. We also included literature addressing countries at all income levels. In order to minimize individual author biases during theoretical sampling, the authors met regularly with each other and with a larger group of subject matter experts to discuss the thematic appropriateness of the included texts.

One hundred and four papers were included in Phase 2, (42 regarding lack of conscientization and normalization of MoW; 26 regarding perceptions of fitness for motherhood; 13 regarding geopolitical and ethnopolitical projects related to fertility; and 23 regarding pressure to achieve quantifiable performance goals). Authors hand coded the included papers and developed these codes into thematic memos.

While extracting and coding data for both Phase 1 and Phase 2, the review team critically appraised each paper using quality appraisal criteria developed by Wallace et al. [31]. Within CIS methodology, it is not necessary to exclude papers based on quality, in part because assessing quality is inherently challenging when the included papers span a variety of disciplines [28]. While we did not exclude papers on the basis of quality, appraisal process did enable us to summarize the limitations of papers that were included.

Recurring limitations that emerged from the quality appraisal process include:

1. Limited generalizability, namely papers that focused on phenomena within a narrow geographic area and/or with a small sample size.
2. A lack of authors’ explicit theoretical or ideological perspective. Specifically, a lack of reflexivity regarding the potential impact of author biases and relative power compared to research participants.

### Phase 3: Data synthesis in the context of programs and interventions

The CIS approach to data synthesis is akin to that of primary qualitative research [26]. Phase 3 involved putting the extraction tool and thematic memos arising from Phase 1 and Phase 2 “in dialogue with one another” to describe the theoretical and empirical learning regarding the drivers identified, and to explore the application of this learning to interventions to address MoW.

To accomplish this, we expanded the thematic coding summaries from Phase 2 to integrate the findings from Phase 1. In order to ensure that we summarized and critically engaged the literature in both phases, we went back and forth between the results to look for convergences, disagreements, and themes to finalize the summaries. We used this analysis to develop analytic propositions, which we present in the discussion section.

To bolster the applicability of our findings for decision-makers in maternal health, we put our discussion regarding policy and program approaches to address MoW in a discrete section. For transparency, we are explicit through the Results section about the basis for our statements, i.e. whether they come from Phase 1 or Phase 2 of the search.

## Results

We present the results in 3 sections–first the result of Phase 1; the results of Phase 2; and then Phase 3, insights regarding ways to address power-related drivers of MoW.

### Phase 1: Overview of scoping review findings

As summarized in S1 Table summarizing the papers reviewed, the papers reviewed in Phase 1 contained original empirical research (quantitative n = 8, qualitative n = 37, mixed-methods n = 5), reviews (n = 6) and commentaries (n = 7). The articles covered a broad geographic range including sub-Saharan Africa (n = 26), Asia (n = 14), Middle East and North Africa (n = 4), Latin America (n = 9), and Eastern Europe (n = 3). Seven papers addressed more than one region. Several countries appear more frequently than others, including India (n = 7), Ethiopia (n = 7), Tanzania (n = 4), and Kenya (n = 4). We only applied the critical appraisal to empirical articles (n = 61). Of these, a small minority could not be assessed using the criteria, such as the paper that was based on ethnographic field notes from multiple projects [32]. The majority of the papers met all criteria (n = 45), while a significant minority did not describe a theoretical basis for the study, and/or the paper did not include any statement or other evidence of reflexivity.

#### Phase 1 papers reviewed

While most papers present drivers of MoW as such, several papers focus on patient attributes that are associated with experiencing MoW (e.g. race, caste, age, and marital status) without explicit attention to causality or structural factors (n = 8). In contrast, some of the papers explicitly aim to explore the drivers of MoW or they address these questions as part of a larger analysis. The power-related drivers of mistreatment in these articles span multiple levels of the social ecological model including intrapersonal (e.g. lack of conscientization), interpersonal (e.g. patient-provider hierarchy), community (e.g. widespread discrimination against indigenous women), organizational (e.g. pressure to achieve performance goals), and law/policy (e.g. lack of accountability for rights violations). Most papers present multi-level interpretations of power dynamics, such as, for example, explorations of how organizational factors shape interpersonal and intrapersonal dynamics.

A small subset of papers focus almost exclusively on intrapersonal factors, namely individual-level expectations of low-quality care and internalized submission (lack of *power to*). These papers state that a lack of individual capacity to identify MoW and to demand respectful care contributes to the persistence of MoW [33, 34]. Examining the organizational and interpersonal levels, several papers describe mistreatment as occurring in contexts where providers, primarily midwives, feel unsupported or even victimized within their professional hierarchies. These providers attempt to assert their power and/or meet professional expectations by ensuring patient “compliance” rather than centering patient needs [35–39]. Interpersonal communication challenges stemming from providers’ and health facilities’ inability to overcome communication barriers relating to language, culture, or social identity are also cited as fostering a climate of MoW [33, 40–42]. Several papers explore if and how birth companionship can change interpersonal dynamics in order to reduce MoW, but they show mixed results [43–46]. Papers suggesting that companionship is or is not protective against MoW present different mechanisms relating to power asymmetries. For example, that companions serve as witnesses or advocates whose presence moderates provider behavior [46], that companions become instruments of MoW [47], or that denial of a birth companion by health workers reflects a power imbalance and constitutes an act of MoW itself [45].

Most articles reviewed describe or at least acknowledge how social and political factors shape organizational and interpersonal dynamics. Government policies, such as the criminalization of abortion, can contribute to stigmatization and discriminatory treatment by providers of patients suspected of having attempted an abortion prior to arrival [43, 48]. Many articles address the intersection of social hierarchies (i.e. anti-indigenous or anti-refugee sentiment) and gender discrimination, and their manifestation in provider attitudes toward certain women reproducing [3, 43, 49–55] or in providers’ facilitation of coerced sex-selective abortion [56]. Another emergent multi-level theme related to the tension between health system organizational performance goals and the provision of patient-centered care. Pressure to achieve targets can increase the likelihood that MoW occurs [35, 56].

While many of the papers invoked concepts implicitly associated with power, such as gender norms and gendered hierarches of power; or simply stated that one actor had power over another; only 9 of the 63 papers included in Phase 1 explicitly applied theory related to power. These included exploration of theories of empowerment and if and how childbearing women’s empowerment was related to the experience of mistreatment [57], as well as applying an intersectionality lens to reveal the ways that internalized stigma makes the transition to motherhood a disempowering experience [58]. Two studies used the theory of street-level bureaucracy, one as a way of explaining health care workers’ unwillingness or inability to make maternity care more culturally accessible to indigenous women [52] and another to describe midwives more generalized rude treatment of patients and their families [36]. Theories of structural violence were invoked by multiple papers [32, 33, 36, 59]; for example to explain normalization, or women’s preference to withstand abuse “in response to restricted options among the structurally deprived” [32, 33]. Drawing on Pierre Bourdieu’s concept of *habitus*, Delgado describes an “authoritarian medical habitus,” which normalizes violence in maternity care, thus denuding any collective response [34]. Finally, Yevoo and colleagues developed new theory to frame the analysis and discussion of the ways women in Ghana provide misinformation regarding their reproductive and medical history as a strategy against healthcare providers’ domination in maternal care decision-making interactions [60].

In sum, with the exception of papers exploring patient attributes that were associated with MoW, the papers identified in our scoping review described ways that power related to MoW at multiple levels of the social ecological model. Many explicitly or implicitly invoked the notion of *power over* labouring women, or lack of *power to* among patients and providers, while relatively few papers applied or explored theories of power.

### Phase 2: Thematic findings

The four themes below are not entirely conceptually or empirically distinct; there is potential overlap and among them and they can be inter-related. We did not apply the critical appraisal used in Phase 1 to these Phase 2 papers as they are from diverse fields, including organizational management and law, and not amenable to a universal quality standard.

#### Lack of conscientization regarding MoW and normalization of MoW

Papers reviewed in Phase 1 identified lack of knowledge about rights and entitlements among labouring women and their families as well as widespread, social normalization of MoW as enablers of MoW; some women, their families, and providers understood MoW to be natural, such that they did not question it [33, 42, 50, 61–63]. The concept of normalization itself is implicitly linked to power, normalization serves to channel expectations about maternity care, and to informally regulate provider and patient behavior [64]. Studies assessed how this plays out in diverse contexts, such as internalized normalization among particular social groups, e.g. homeless women [61]. Normalization and lack of rights knowledge manifests in differential rates of reporting about whether or not the patient experienced mistreatment. For example, two studies found that women who were more educated were more likely to report having experienced mistreatment, which the authors attributed to better knowledge about rights and higher expectations about health services among more educated women [65, 66].

Moreover, many Phase 1 studies pointed out that obstetric services were organised around the needs and logic of the health facility, rather than on those of labouring women, contributing to conditions that directly undermine respectful maternity care, such as lack of privacy, and, directing provider attention to organizational priorities rather than to patient care. This prioritization of organizational needs rather than patient needs is a manifestation of normalization, insofar as the primacy of organizational needs is largely unquestioned [34]. This undercuts quality of care, including respectful maternity care [32, 36–39].

In Phase 2, we sought to understand how normalization and lack of entitlements knowledge were addressed in the respectful maternity care literature, as well as in the public health and human rights literature more broadly, and the implications for change.

Some researchers explore how the prioritization of facility needs normalizes MoW, shaping both provider and patient expectations and perceptions of the health care encounter. Provider practices that are described as MoW may be deeply engrained, such that providers do not consider them to be problematic, and consider them to be logical in the context. Examples include paying more attention to women in labour to whom the provider has a connection or who can pay [67–69], not sanctioning fellow midwives for mistakes [67], and yelling or slapping women who are perceived as not pushing hard enough [66, 67, 69, 70]. Indeed, compelling a woman to push may be routinized as a way of providing good care to avoid obstetric emergencies, particularly during the second stage of labour [43, 50]. Normalization of MoW is particularly likely to arise in contexts where performance according to the elaborated standards is impossible, given human and other resource scarcity [58, 64, 69, 70]. Against this backdrop, deviations from standards of quality are unavoidable and expected. MoW is not only linked to resource scarcity however, with some research, for example, finding that expectations of MoW among labouring women leads them to submit to increasingly medicalized childbirth [71]. More broadly, the normalization of MoW has been described as a manifestation of widespread normalization of violence against women in society [72, 73].

Just as providers may not question or examine some behaviors that comprise MoW, for their part, patients may expect them and feel they have little power to change them; this was evidenced in the studies finding comparatively few women reporting having experienced MoW in Phase 1. Some qualitative analyses suggest that patients report high levels of satisfaction in part because they are reluctant to evaluate the care received critically, particularly patients of lower social, economic, reproductive, or marital status [74]. This reluctance stems from feeling dependent on providers and lack of power within a biomedical setting [72, 74]. In other words, patient assessment of quality of care is influenced by broader unjust power relations [75].

Given that normalization is embedded in health system and broader social dynamics, merely communicating rights and entitlements to pregnant women and their communities is insufficient. Yamin notes that human rights frameworks focus on human beings as autonomous individuals, without fully acknowledging that social and political relations comprise a broader structure that limits health worker, patient, and community agency [75, 76]. Some researchers describe MoW as an active process; it is a way that providers blame patients for their situation, actively asserting their own social superiority or deflecting blame in punitive environments [74]. In this context, women seeking to claim their rights and entitlements can face significant risk and retaliation for doing so [76].

#### Perceptions regarding fitness for motherhood

Several papers in Phase 1 addressed provider perceptions that women who fail to meet social expectations of ideal motherhood deserve mistreatment. While notions of the “ideal mother” varied by context, in Phase 1, providers perceived women as problematic mothers on the basis of attributes such as disability, social class, age, and marital status [16, 50, 58, 77]. Unfitness for motherhood has multiple dimensions: women assumed to be materially or physically unable to provide for their child’s welfare [78] or women assumed to be morally unfit to do so, such as single mothers [58].

In Phase 2 we examined diverse literature from midwifery, sociology, disability studies, and public health journals to shed light on the ways that problematic motherhood can be constructed and enforced in health care settings.

The ideal mother in different contexts includes attributes relating to class, age, race, ability, or other forms of privilege [79, 80]. An intersectional analysis exposes the ways that interrelated axes of marginalization can contribute to judgements regarding motherhood and subsequent mistreatment [14, 72, 81]. For example, adolescent mothers of low socioeconomic status can face discriminatory treatment on the basis of both class and age [58]; similarly, mothers who are both indigenous and chronically ill can face concurrent discrimination [82].

The disability studies literature has a rich strain of research that systematically focuses on the way that people with disabilities themselves describe and analyze their experiences. This systematic approach adds particular value to a discussion about MoW within the global health field, where patient experiences are still often relegated to “demand side” concerns, such as promoting “care seeking” [83]. In contexts where gender norms elevate motherhood as a societal ideal, “achieving” motherhood can represent a “status passage” to normality for women with disabilities or who are otherwise marginalized [84, 85]. Some providers, in contrast, believe that women with disabilities do not or should not exhibit the sexuality, assertiveness, or general skills expected of women participating in marriage and childbirth [86]. In a study of prenatal education among women with disabilities, women reported receiving poor quality or insufficient information about their pregnancy because they believed that nurses doubted their capacity for decision-making and "proper" motherhood [87].

Public health literature describes other forms of institutional responses to “deviant” motherhood. Teen pregnancy is one example, as teen pregnancy and parenting are constructed as “social problems” because they make visible young women’s sexuality and display a form of motherhood at odds with provider expectations of maternal maturity and selflessness–a difficult dynamic for young mothers to confront during healthcare utilization [88, 89]. Similarly, maternal substance use may be constructed societally as an individual moral deficit and departure from expectations of purity, a framework which undercuts the provision of evidence-based, non-judgmental treatment [79, 89].

The global health literature shows how these deeply held prejudicial beliefs can result in the exercise of provider power over the patient’s body and reproductive life. For example, providers performing non-consented sterilization of HIV positive women may believe that HIV positive women are poor decisionmakers who are undeserving of motherhood, demonstrating the power wielded by predominantly male doctors who presumably do not fear punishment [90, 91].

Much of the empirical research related to perceptions of motherhood reviewed in Phase 1 simply notes patient attributes and documents MoW stemming from providers’ prejudicial beliefs about people holding those attributes. The broader social science literature provides additional insight into how such prejudicial beliefs–indeed, how the very categories to which these prejudices attach–are formed and sustained. For example, the literature on racial formation in the United States demonstrates how, historically, those with societal power have used their control over laws, social norms, and discursive practices to define “Black” and “white” and to situate those categories in a hierarchy that values Black and white people differently. One way this devaluing and the mistreatment that accompanies it are sustained is by asserting that the hierarchy is natural, inevitable and unchangeable [88]. In this way, power disguises its own operation.

#### Geopolitical and ethnopolitical projects related to fertility

This understanding of how power works to create and enforce racial categories has had particular resonance in explaining mistreatment of Black women in the United States during pregnancy and childbirth. Women’s reproduction was central to the institution of slavery and to the operation of Jim Crow laws that enforced segregation and discrimination after the abolition of slavery in the 1860s until the Civil Rights laws of the 1960s. Since that time, scholars have demonstrated how laws, policies and practices that are neutral on their face are implemented in ways that continue surveillance and policing of Black women’s reproduction and motherhood [89]. For example, many health workers and hospitals in the US have aided this dynamic by disproportionately surveilling and reporting Black women’s drug use during pregnancy, contributing to higher levels of incarceration of Black women [90]. Incarcerated women, who are disproportionately Black, have routinely been subjected to mistreatment during childbirth including shackling during labor and traumatic removal of their newborns into the welfare system [91].

This phenomenon is not limited to the United States. Disproportionate mistreatment of darker-skinned incarcerated women during hospital-based childbirth has been reported in Brazil, regarded by many as a mechanism to uphold racial and class hierarchies [92, 93].

Several papers in Phase 1 identify MoW as driven in part by geopolitical and ethnopolitical projects that infuse provider perspectives on reproduction, including regarding women’s fitness for motherhood. Yasmine and Moughalian frame Lebanese health worker mistreatment of pregnant Syrian refugees within the context of prevalent beliefs that Syrian refugees are to blame for Lebanon’s social and economic problems [94]. Chattopadhyay suggests that the legacy of British colonialism and citizenship hierarchies in India influence maternal health providers’ beliefs that Bengali Muslims are at fault for “excess fertility” [95]. Similarly, based on qualitative interviews, Mohammadi and colleagues conclude that the politicization of Afghan refugee status in Iran contributes to discrimination by health workers during severe maternal morbidity events [41].

In Phase 2, we explored examples in which facility-based mistreatment occurs implicitly or explicitly in the name of geo- and ethnopolitical state objectives. We identified theoretical and empirical work on “reproductive governance” exploring how social and economic institutions and law and policy operate to influence directly or indirectly reproductive lives of individuals in service of political aims [96].

Several articles demonstrated health workers’ power over undocumented pregnant women. Afghan refugees in Iran who lacked documented status reported fears that health workers would facilitate their deportation and arrest while obtaining prenatal care, a phenomenon related to political tensions between Iranians and Afghan refugees [97]. Healthcare workers can also be tasked with enforcing immigration policies, deterring some patients from seeking care, and making the healthcare encounter fraught with stress for those who do [98]. For instance, German laws require public health system employees to report undocumented patients to police or foreign authorities, so pregnancy represents a particularly vulnerable period for undocumented women [98, 99].

#### Pressure to achieve quantifiable performance goals

Several Phase 1 papers indicated that performance-based financing, politically salient health coverage goals, and other strategies for performance improvement and management could draw provider attention to the services being measured and/or incentivized, at the expense of unmeasured or unincentivized quality of care issues, including respectful maternity care [36, 60, 100]. Activities typically incentivized and/or included in global goals include service provision or coverage, such as institutional delivery, or care provided, such as two doses of intermittent preventive treatment for malaria (IPT2) during antenatal care [101]. In Phase 2, we explored the foundational literature on performance-based financing and other efforts to aggressively manage quantifiable outcomes in the global health, business, and public administration literature, as well as critiques of these schemes as manifestations of power in global health.

Some social science research concludes that aggressive performance management and improvement efforts in global health instrumentalizes labouring women as means to an end, reinforcing interpersonal and organizational power dynamics and intrapersonal normalization of MoW by undercutting the autonomy of women in labour [83, 102, 103]. Women giving birth in a health facility (as opposed to at home) or surviving childbirth is counted as a success; their subjective experience or the maintenance of their dignity is not relevant in most models of performance management [83, 104], such that what is not measured does not count [101, 104–107]. Midwives in Benin interviewed as part of research on severe obstetric events reported that many development projects encouraged a “semblance of quality of care rather than actually producing it” [108]. Such instrumentalization can also directly contribute to MoW, as health care workers use punitive tactics–ranging from verbal reprimands, to hitting, to imposing fines, to make sure that labouring women do what the providers believe is required to meet relevant goals [70, 101, 109].

We found corollaries in the business and public administration literature, where several studies showed that incentives and aggressive goals can lead to unethical behavior that is inconsistent with the mission of the organization in question, as employees receive signals from management that goal attainment is valued above all else [110]. This “mission inconsistent” behavior can become an embedded, informal norm over time [110]. There is some evidence for similar dynamics in the global health literature. For example, a study in the Democratic Republic of Congo assessed the aftermath of Performance-Based Financing (PBF) and found that providers in facilities where PBF had previously been in place expressed lower motivation and worse relationships with the community than providers in facilities that had never implemented PBF programs. This suggests that the program had “crowded out” pro-social motivation [111].

The business and public administration literature evinces near consensus that in general, financial incentives in particular do not advance the objective of the organization applying them [106]. In the context of global health, particularly maternal and child health, research in some countries finds that pay-for-performance leads to positive outcomes in health service quality and coverage [102] but a 2020 Cochrane review found that pay-for-performance “may have slight positive impacts on the majority of health outcomes appraised against a pure control or standard care; however, when compared against other interventions such as enhanced financing, limited to no impacts were identifiable” [112]. The pro-social motivation of health care workers, such as their commitment to patient-centered care; their own goals; and whether or not those pro-social engagements and personal goals are reflected in performance management and improvement efforts appear key to shaping outcomes [101, 102, 109, 113, 114].

Zooming out, aggressive performance management and improvement efforts have been described as a symptom of a broader trend of technocratic narrowing and focus on the “numbers game” that has come to dominate maternal health funding and agenda setting. These priorities may reflect the material and discursive power of key actors in the wealthy (donor) countries who have their own approach that are not necessarily concordant with those of the people or the governments in countries where these programs are to be implemented [115]. For this reason, many social scientists have highlighted the need to examine the impact of such efforts on worker motivation and the quality of social relations, since these have implications for health sector performance over the long term [102].

### Phase 3: Approaches to addressing power

Phase 2 thematic searches yielded several streams of literature relating to ways that power dynamics could be shifted to promote RMC. We analyze that literature here in the context of the drivers we selected, i.e. lack of conscientization and normalization of MoW, perceptions regarding fitness for motherhood, geo- and ethno-political projects related to fertility and pressure to achieve quantifiable performance goals. Many of these operate at multiple levels of the social ecological model. We summarize key findings in Table 2.

**Table 2:** Literature on ways that power dynamics could be shifted to promote RMC.

Key related drivers of MoW	Approach to promoting RMC	Description
Lack of conscientization, normalization, perceptions regarding fitness for motherhood, geo and ethno-political projects	Human Rights Education	Training to promote the values and commitments in international and/or national human rights law
	Conscientization	Facilitate marginalized individuals’ developing a critical awareness of their own social reality through reflection and action. Intended to spur collective action
Normalization, perceptions regarding fitness for motherhood, pressure to achieve quantifiable public health goals	Dialogic approaches	Dialogical approaches bring together service providers and service users to discuss quality concerns in part by bringing the experiences of the marginalized into the discussion
	Reducing provider bias	“Early intervention” programs that disrupt hidden curricula:
		• programs to identify and change perceptions that obstetric violence is normal among health science students
		• values clarification workshops and other change efforts that entail significant provider reflection
		• stronger focus on reflective practice in medical curricula, and/or the inclusion of social sciences and the humanities in medical education
		• implicit bias training for practicing clinicians
		• structural competency trainings that ground anti-bias training in the context of broader social histories and modifiable power dynamics
		• training health workers as advocates for policy change for reproductive health

Research and program experiences in human rights education and conscientization offer insight into efforts to address individual beliefs and social norms underlying lack of conscientization, normalization, perceptions of fitness for motherhood, and geo- and ethno-political projects. Human rights education entails training to promote the values and commitments enshrined in the international human rights system. Programs based in conscientization and human rights education can address service providers and communities, working from theories of change premised on the notion that education about rights and entitlements and facilitated reflection on one’s own political and social position can spur reflection and action [2, 14, 16, 22–119]. Conscientization in particular envisions collective action, stemming from the assumption that individual actions are insufficient to transform power dynamics that oppress collectives. In the health context, this includes empowering patients and communities with biomedical information that partly redresses the patient/provider power imbalance, such as the movement for treatment literacy within HIV [120], and in the context of maternal health specifically, birth preparedness programs that have an explicit empowerment component [65, 121–123].

Dialogical programs convene both service providers and service users, and seek to bring the experiences of the marginalized into mainstream discussion, such as stories about experiences of poor or discriminatory treatment by health providers [116, 117, 124]. This can be accomplished using various approaches, ranging from human rights education and conscientization to Participatory Action Research [125], developing political capabilities among those communities that disproportionately experience MoW [126], the utilization of formalized processes for civil society engagement in governmental priority setting and program evaluation [76], soliciting in-depth feedback from women following labour and delivery [74], and social accountability or legal empowerment approaches [127, 128].

However, the literature on professional norms and “hidden curricula” suggests that dialogue-oriented efforts are limited by whether and to what extent providers feel they should be accountable to poor communities [71, 76]. Feelings of professional obligation are formed early and constantly reinforced; these formative values are often tacit and unacknowledged. Inaccurate or informally communicated curricula normalize provider assumptions that contribute to poor quality care [83, 129] such as, for example, widespread false viewpoints about biological differences between Black and white patients’ pain tolerance among US medical students [129, 130]. While much of the research on “hidden curricula” has been done in North America [131, 132], the concept focuses our attention on the effects of social structures, organizational priorities, and culture and the resultant practices that are “antithetical to humanism” [131, 132], and transmitted during medical education, though not part of the formal curriculum [133]. These result in socially driven–rather than clinically driven–judgements, such as which patients are worthy of quality care or deserving of being attended to immediately [133].

Some programs seeking to change engrained and actively perpetuated values intervene early, such as programs to identify and change perceptions that obstetric violence is normal among health science students [134], values clarification workshops [135], and other change efforts that entail significant provider reflection [136]. Researchers who have conduced reviews on hidden curricula suggest that changes in medical curricula might include a stronger focus on reflective practice, and/or the inclusion of social sciences and the humanities in medical education [137, 138].

Training providers on the ways implicit biases shape patient/provider interactions is a common approach. By focusing on largely unconscious behaviors, such training may offer a relatively inoffensive awareness-raising opportunity for provider reflection on their role in upholding inequality [139–142]. However, some researchers and advocates assert that by assuming that biases are manifest primarily in individual, passive behavior, as opposed to structures, implicit bias interventions fall short of a comprehensive power diagnosis, and may put the onus for change on frontline actors rather than the individuals and institutions with the most power [143–146].

Other clinician training interventions, such as structural competency trainings, attempt to ground anti-bias training in the context of broader social histories and modifiable power dynamics. These trainings examine multiple levels of the social ecological model, such as training providers to recognize health care institutions as structures that influence power dynamics [147], or training health workers to be advocates for policy change, such as to alter policies that criminalize or stigmatize certain patient groups [143–145]. Relatedly, clinician interventions utilizing reproductive justice frameworks train and empower clinicians to intervene during instances of mistreatment or to serve as broader societal advocates for reproductive justice [74, 148–150]. Reproductive justice, as envisioned by Black feminist advocates in the US, includes the key tenets of rejecting population control/eugenics; centering marginalized groups; recognizing intersecting forms of oppression; linking theory, strategy, and practice; and commitment to shifting power [148].

Literature regarding ways to address other power-related drivers, such as pressure to achieve quantifiable performance-related targets, is more limited and diffuse. This is particularly the case where policies themselves are drivers, and one obvious solution is to end the policy. However, even in the context of debates regarding the presence or absence of a given policy, the preceding discussion regarding ways of shifting deeply embedded norms is instructive, as policies often reflect norms. In other words, norms change is one route to addressing the drivers of the drivers.

## Discussion

Phase 1 revealed the ways that power has been conceptualized and operationalized in academic explorations of MoW. In Phase 2, we pursued prioritized thematic lines of enquiry to explore the theories and evidence regarding under-explored, power-related drivers of MoW, and in Phase 3, we synthesized the papers, with a particular focus on interventions to promote RMC. The wide-ranging findings coalesce in several key meta-themes, each with their own evidence-base for action. We present these meta-themes as analytic propositions emerging from our review, and, consistent with the notion that research on power points us to “drivers of the drivers,” include some intervention-relevant insights for further exploration.

### Mistreatment of women is a manifestation of broader social norms, moderated by stigma and prejudicial beliefs of individual providers

This statement is axiomatic, but our findings underlined the ways that stigma and discrimination in particular shape MoW; they are manifest in providers’ power over labouring women, as well as in women’s beliefs and capacity to claim rights (i.e. power to). (A companion paper in this Series presents a mixed-methods systematic review of strategies to reduce stigma and discrimination in sexual and reproductive healthcare settings [149]) Moreover, stigma and discrimination are driven by a variety of factors, including state geo- and ethno-political objectives, harmful community norms, and provider prejudices regarding fitness for motherhood. In contexts where pressure to achieve objectives is high and resources are scarce, providers may feel that differential treatment of patients is unavoidable and thus mete out worse treatment to lower status patients.

### Health systems may be designed and operated in a way that perpetuates MoW

Within the broader field of research on power, critical theory centers the concerns of people who lack *power to* [150]; in Phase 2, we saw this in the disability studies literature. Indeed, this orientation is reflected in ethnographic work focusing on women’s experiences of MoW [74, 151]. Reframing health systems analysis to position labouring women’s experiences as the conceptual starting point, rather than health system performance or operations as the starting point, may facilitate research and policy that promotes respectful maternity care. As evidenced by the research on aggressive performance management of quantifiable health goals, approaches grounded in limited definitions of health systems performance may elide the ways programs exacerbate MoW, as well the inter-related dynamics that shape provider satisfaction and motivation. In addition, refocusing discussions about addressing MoW on patient experiences points the way to structural changes that go beyond seeking to change provider beliefs. However, approaches that center patient experiences should also be attentive to the role played by normalization and social risk for rights claiming among marginalized women.

### Meaningfully shifting power dynamics requires multi-level strategies

The descriptive articles reviewed showed how power was manifest at different socio-ecological levels. While MoW may be experienced by individuals, the phenomenon is largely not shaped at the individual level. Yet, many extant programs seek to change beliefs among providers on an individual basis, such as through trainings. Given that the evidence base reveals a multi-level challenge, we focused our assessment of interventions on multi-level solutions, including some that sought to enhance *power to* among those with less power, and some that sought to generate and mobilize collective power to affect change. Some of what we found seeks to instigate multi-level change by working with affected individuals, such as conscientization, dialogic approaches, and reproductive justice informed training with providers. Research on structural change in the HIV field shows the key role played by social movement activism, or “people power,” which can create the conditions for programs and interventions focused on the health system to succeed [119].

This paper synthesizes several streams of social science research, and accordingly certain factors should be considered while interpreting the results. First, given that this paper is not a systematic review of how power may influence maternity care, we did not review articles on topics that may be relevant, such as religious fundamentalism. Second, power is not a straightforward concept. As a team, we established internal guidelines to decide which papers were implicitly invoking power, and to assess if and how papers using the same power-related terms, such as “gendered power relations” or discrimination, interpreted those constructs in similar ways. We were consistent in our decision-making, but a different team might make different decisions. Finally, power is a wide-ranging concept and in the context of writing a synthesis, it is challenging to strike a balance between describing and analyzing drivers of the drivers and being adequately parsimonious to shed light on modifiable factors. Power is not a parsimonious construct, but its encompassing nature allows us to see patterns and themes in the drivers of MoW. In other words, we zoom out to understand the ways that power dynamics at all levels of the socio-ecological model influence and reinforce each other. The fact that intrapersonal and interpersonal dynamics are so profoundly shaped by community, organizational, and broader political factors shows the necessity of systems change to address MoW. Future research might explore the how of such systems change. A key challenge will be the fact that some efforts to address upstream power-related drivers of MoW might not be designed or described as efforts to address MoW at all, such as efforts to recruit and train more woman physicians, or successful efforts by stigmatized minority groups to reduce discrimination.

This paper has several limitations. While we provide significant detail about our methods, different authors might have designed a different process, yielding different insights. In addition, our synthesis and analysis are limited to the content of the papers we included, and by the weaknesses of these papers. The quality review indicated a significant minority of the empirical papers reviewed in Phase 1 lacked a theoretical perspective, undermining our ability to draw out theories in our analysis, although we recognize that a lack of explicit theoretical perspective does not mean that the authors did not have an epistemological starting point. Finally, this paper was limited to a peer-reviewed literature, but the inclusion of reports or other types of grey literature might provide good insight into women’s perspectives, or other elements that are less well-addressed by the peer-reviewed literature.

## Conclusion

Our analysis seeks to connect the dots regarding power-related drivers of MoW among the evidence base specific to MoW, the broader social sciences literature, and evaluations and critiques of evaluations that seek to alter power asymmetries or to mobilize power in the service of human rights promotion.

A nuanced diagnosis of the ways the current distribution of power engenders MoW is important to any theory of change to reduce MoW; an assessment of the strengths and weaknesses of various approaches that have been tried can shed light on how the distribution of power might be shifted. The extant peer-reviewed literature on MoW provides a firm foundation for further research on the relationship between power and MoW at various levels of the social ecological model. However, because some of this research is not theory-based and because of the depth of the challenge, further programs and health systems research are needed to flesh out how a multi-level effort could meaningfully address power to reduce MoW and promote respectful care. To do this, a multi-level and multi-faceted health systems approach with the engagement of different stakeholders, including women, community, health workforce, and policy-makers will be critical.

## Supporting information

S1 Table Phase 1 papers reviewed. (DOCX)
