# Supplementary material for: A critical interpretive synthesis of power and mistreatment of women in maternity care
Source: PLOS Glob Public Health. 2023 Jan 30;3(1):e0000616. doi: 10.1371/journal.pgph.0000616 (PMC10021192; doi:10.1371/journal.pgph.0000616)
Supplement: S1 Table — (DOCX) [file pgph.0000616.s001.docx]

| **Author(s)** | **Title** | **Country** | **Level of power dynamics** | **Drivers** | **Study type** |
| --- | --- | --- | --- | --- | --- |
| (Marques de Aguiar, J; d'Oliveira, A.F.P.L.; Schraiber, L.B.) | Institutional violence, medical authority, and power relations in maternity hospitals from the perspective of health workers | Brazil | interpersonal, community, organizational | Fitness for motherhood; stigma and discrimination; normalization of MoW | Qualitative |
| (Akasreku, B.D.; Habib, H.; Ankomah, A) | Pregnancy in Disability: Community Perceptions and Personal Experiences in a Rural Setting in Ghana | Ghana | interpersonal, community, organizational | Communication issues, Perceptions around fitness for motherhood, Stigma and discrimination | Mixed methods |
| (Amroussia, N.; Hernandez, A.; Vives-Cases, C.; Goicolea, I.) | “Is the doctor God to punish me?!” An intersectional examination of disrespectful and abusive care during childbirth against single mothers in Tunisia | Tunisia | intrapersonal, interpersonal, community, organizational | Lack of conscientization; Fitness for motherhood; stigma and discrimination; normalization of MoW | Qualitative |
| (Ansari, M.S.; Manzoor, R.; Siddiqui, N.; Ahmed, A.M.) | Access to comprehensive emergency obstetric and newborn care facilities in three rural districts of Sindh province, Pakistan | Pakistan | Interpersonal, community, organizational | Health systems that do not earn users' trust, Stigma and discrimination | Mixed methods |
| (Aziz, A.; Khan, F.A.; Wood, G.) | Who is excluded and how? An analysis of community spaces for maternal and child health in Pakistan | Pakistan | intrapersonal, interpersonal, community, organizational | Lack of conscientization; Providers feel unsupported within professional hierarchies; Communication issues; Normalization of MoW; Stigma and discrimination | Qualitative |
| (Baranowska, B.; Doroszewska, A.; Kubicka-Kraszyńska, U.; Pietrusiewicz, J.; Adamska-Sala, I.; Kajdy, A.; Sys, D.; Tataj-Puzyna, U.; Bączek, G.; Crowther, S.) | Is there respectful maternity care in Poland? Women’s views about care during labor and birth | Poland | interpersonal | Lack of conscientization; communication issues; Stigma and discrimination | Quantitative |
| (Bohren, M.A.; Hunter, E.C.; Munthe-Kaas, H.M.; Souza, J.P.; Vogel, J.P.; Gülmezoglu, A.M.) | Facilitators and barriers to facility-based delivery in low- and middle-income countries: a qualitative evidence synthesis | Multiple (review) | interpersonal, community, organizational | Stigma and Discrimination; Pressure to achieve quantifiable public health goals | Systematic Review |
| (Bohren, M.A.; Mehrtash, H.; Fawole, B.; Maung, T.M.; Balde, M.D.; Maya, E.; Thwin, S.S.; Aderoba, A.K.; Vogel, J.P.; Irinyenikan, T.A.; Adeyanju, A.O.; Mon, N.O.; Adu-Bonsaffoh, K.; Landoulsi, S.; Guure, C.; Adanu, R.; Diallo, B.A.; Gülmezoglu, A.M.; Soumah, A; Sall, A.O.; Tunçalp, Ö) | How women are treated during facility-based childbirth in four countries: a cross-sectional study with labour observations and community-based surveys | Ghana, Guinea, Myanmar, Nigeria | Interpersonal | Fitness for motherhood; Stigma and discrimination | Mixed methods |
| (Bradley, S.; McCourt, C.; Rayment, J.; Parmar, D) | Disrespectful intrapartum care during facility-based delivery in sub-Saharan Africa: A qualitative systematic review and thematic synthesis of women's perceptions and experiences | Multiple (review) | Interspersonal, Organizational, Law/Policy | Providers feel unsupported within professional hierarchies; Policies as drivers of discrimination; Pressure to achieve quanitifiable public health goals | Systematic review and meta synthesis |
| (Castro, A.) | Witnessing Obstetric Violence during Fieldwork | Mexico, Dominican Republic | interpersonal, interpersonal, community, organizational, law and policy | Lack of conscientization, providers feel unsupported within professional hierarchies; normalization of MoW; Stigma and discrimination; Pressure to achieve quantifiable public health gaols | Commentary |
| (Castro, A.; Savage, V.) | Obstetric Violence as Reproductive Governance in the Dominican Republic | Dominican Republic | interpersonal, community, organizational | Normalization of MoW, Stigma and Discrimination; Perceptions around fitness for motherhood; Geopolitical or ethnopolitical projects | Qualitative |
| (Castro, A.; Savage, V.; Kaufman, H.) | Assessing equitable care for indigenous and afrodescendant women in Latin America | Multiple (review) | Interpersonal, Community, Organizational | Stigma and Discrimination; Perceptions regarding fitness for motherhood; Providers feel unsupported within professional hierarchies; Geopolitical or ethnopolitical projects | Literature review |
| (Chattopadhyay, S.) | The shifting axes of marginalities: the politics of identities shaping women’s experiences during childbirth in Northeast India | India | Interpersonal, community, organizational | Providers feel unsupported within professional hierarchies; Pressure to achieve quanitifiable public health goals; Stigma and discrimination; Social and professional location of providers signifies something about their behavior toward women; Geopolitical and ethnopolitical projects | Commentary |
| (Delgado, X.B.M.; Enciso Chaves, L.V.; Yepes, C.E.) | Neither Medicine Nor Health Care Staff Members Are Violent By Nature: Obstetric Violence From an Interactionist Perspective | Colombia | interpersonal, community, organizational | Normalization of MoW, Helath systems that do not earn users' trust; Pressure to achieve quanitifiable public health goals; Poor regulation of private pecuniary motives | Qualitative |
| (Dey, A.; Shakya, H.B.; Chandurkar, D.; Kumar, S.; Das, A.K.; Anthony, J.; Shetye, M.; Krishnan, S.; Silverman, J.G.; Raj, A.) | Discordance in self-report and observation data on mistreatment of women by providers during childbirth in Uttar Pradesh, India | India | Interpersonal, Organizational | Normalization of MoW; Perceptions of fitness for motherhood | Qualitative |
| (Diamond-Smith, N.; Treleaven, E.; Murthy, N.; Sudhinaraset, M.) | Women’s empowerment and experiences of mistreatment during childbirth in facilities in Lucknow, India: results from a cross-sectional study | India | interpersonal, community, organizational | Internalized submission, Lack of conscientization, Normalization of MoW, Companionship during institutional births | Quantitative |
| (Dzomeku, V.M.; Boamah Mensah, A.B., Emmanuel Kweku; A.Pascal; Lori, J.R.; Donkor, P.) | “I wouldn’t have hit you, but you would have killed your baby:” exploring midwives’ perspectives on disrespect and abusive Care in Ghana | Ghana | interpersonal, oranizational | Normalization of MoW; Perceptions of fitness for motherhood; Stigma and discrimination | Qualitative |
| (Gebremichael, M.W.; Worku, A.; Medhanyie, A.A.; Berhane, Y.) | Mothers’ experience of disrespect and abuse during maternity care in northern Ethiopia | Ethiopia | Interpersonal; Community | Internalized submission, Normalization of MoW, Stigma and discrimination | Quantitative |
| (Gebremichael, M.W.; Worku, A.; Medhanyie, A.A.; Edin, K.; Berhane, Y.) | Women suffer more from disrespectful and abusive care than from the labour pain itself: a qualitative study from Women’s perspective | Ethiopia | Interpersonal, Organizational | Normalization of MoW; Providers feel unsupported within professional hierarchies; Health systems that do not earn users' trust | Qualitative |
| (Gebreyesus, H.; Mamo, A.; Teweldemedhin, M.; Gidey, B.; Hdush, Z.; Birhanu, Z.) | Experiences of homeless women on maternity health service utilization and associated challenge in Aksum town, Northern Ethiopia | Ethiopia | Interpersonal; Community | Stigma and Discrimination; Perceptions regarding fitness for motherhood | Qualitative |
| (Behboodi-Moghadam, Z.; Khalajinia, Z.; Nasrabadi, A-R.N.; Mohraz, M.; Gharacheh, M.) | Pregnancy through the Lens of Iranian Women with HIV: A Qualitative Study | Iran | Interpersonal; Community | Stigma and Discrimination; Perceptions regarding fitness for motherhood | Qualitative |
| (Gómez‐Suárez, M.; Mello, M.B.; Gonzalez, M.A.; Ghidinelli, M.; Pérez, F.) | Access to sexual and reproductive health services for women living with HIV in Latin America and the Caribbean: systematic review of the literature | Multiple (review) | Interpersonal | Perceptions regarding fitness for motherhood; Stigma and discrimination | Systematic Review |
| (Gourlay, A.; Wringe, A.; Birdthistle, I.; Mshana, G.; Michael, D.; Urassa, M.) | “It Is Like That, We Didn't Understand Each Other”: Exploring the Influence of Patient-Provider Interactions on Prevention of Mother-To-Child Transmission of HIV Service Use in Rural Tanzania | Tanzania | interpersonal | Stigma and discrimination; Health systems that do not earn users' trust | Qualitative |
| Hameed, Waqas; Avan, Bilal Iqbal | Women's experiences of mistreatment during childbirth: A comparative view of home- and facility-based births in Pakistan | Pakistan | Interpersonal | Lack of conscientization; Stigma and discrimination; Communication issues; Normalization of MoW | Quantitative |
| (Heerink, F.; Krumeich, A.; Feron, F.; Goga; A.) | “We are the advocates for the babies” - understanding interactions between patients and health care providers during the prevention of mother-to-child transmission of HIV in South Africa: A qualitative study. | South Africa | individual/interpersonal | Lack of conscientization; Providers feel unsupported within professional hierarchies; Normalization of MoW; Stigma and discrimination | Qualitative |
| (Jardim, D.M.B.; Modena, C.M.) | Obstetric violence in the daily routine of care and its characteristics | Multiple (review) | interpersonal, community, organizational | Lack of conscientization; Health systems that do not earn users' trust; Normalization of MoW; Non-enforcement of laws, lack of accountability; Stigma and discrimination; Social and professional location of the providers | Review |
| (Kendall, T.; Albert, C.) | Experiences of coercion to sterilize and forced sterilization among women living with HIV in Latin America | El Salvador, Honduras, Mexico, Nicaragua | interpersonal | Stigma and discrimination | Mixed methods |
| (Lambert, J.; Etsane, E.; Bergh, A-M.; Pattinson, R.; van den Broek, N) | ‘I thought they were going to handle me like a queen but they didn't’: A qualitative study exploring the quality of care provided to women at the time of birth | South Africa | Interpersonal, Organizational | Lack of conscientization; Providers feel unsupported within professional hierarchies; Helath systems that do not earn users' trust; Normalization of MoW | Qualitative |
| (LeMasters, K.; Wallis, A.B.; Chereches, R.; Gichane, M.; Tehei, C.; Varga, A.; Tumlinson, K.) | Pregnancy Experiences of Women in Rural Romania: Understanding Ethnic and Socioeconomic Disparities | Romania | interpersonal, organizational, community | Communication issues; Non-enforcement of law, lack of accountability; Companionship during institutional births; Stigma and discrimination; | Qualitative |
| (Llamas, A.; Mayhew, S.) | “Five hundred years of medicine gone to waste”? Negotiating the implementation of an intercultural health policy in the Ecuadorian Andes | Ecuador | interpersonal, organizational | Communication issues; Stigma and discrimination | Qualitative |
| (Madhiwalla, N.; Ghoshal, R.; Mavani, P.; Roy, N.) | Identifying disrespect and abuse in organisational culture: a study of two hospitals in Mumbai, India | India | Interpersonal; Organizational | Providers feel unsupported within professional hierarchies; Health systems that do not earn users' trust; Normalization of MoW | Qualitative |
| (Madula, P.; Kalembo, F.W.; Yu, H.; Kaminga, A.C.) | Healthcare provider-patient communication: a qualitative study of women’s perceptions during childbirth | Malawi | interpersonal | Communication issues; Stigma and discrimination | Qualitative |
| (McCallum, C.; Menezes, G.; dos Reis, A.P.) | The dilemma of a practice: experiences of abortion in a public maternity hospital in the city of Salvador, Bahia | Brazil | Interpersonal; Organizational; Community | Health systems that do not earn users' trust; Stigma and discrimination; Policies as drivers of stigma | Qualitative |
| (Mohammadi, S.; Carlbom, A.; Taheripanah, R.; Essén, B.) | Experiences of inequitable care among Afghan mothers surviving near-miss morbidity in Tehran, Iran: a qualitative interview study | Iran | Interpersonal, Organizational, Community | Perceptions regarding fitness for motherhood; Stigma and discrimination; Geopolitical or ethnopolitical projects | Qualitative |
| (Morgan, R.; Tetui, M.; Muhumuza Kananura, R.; Ekirapa-Kiracho, E.; George, A.S.) | Gender dynamics affecting maternal health and health care access and use in Uganda | Uganda | Interpersonal | Stigma and discrimination; Perceptions regarding fitness for motherhood | Qualitative |
| (Moyer, C.A.; Adongo, P.B.; Aborigo, R.A.; Hodgson, A.; Engmann, C.M.) | ‘They treat you like you are not a human being’: Maltreatment during labour and delivery in rural northern Ghana | Ghana | interpersonal, organizational | Providers feel unsupported within professional hierarchies; Social and professional location of providers | Qualitative |
| (Mselle, L.T.; Kohi, T.W.; Dol, J.) | Humanizing birth in Tanzania: a qualitative study on the (mis) treatment of women during childbirth from the perspective of mothers and fathers | Tanzania | Interpersonal; Organizational | Health systems that do not earn users' trust; Non-enforcement of laws, lack of accountability; Poor regulation of private pecuniaray motivations | Qualitative |
| (Oluoch-Aridi, J.; Smith-Oka, V.; Milan, E.; Dowd, R.) | Exploring mistreatment of women during childbirth in a peri-urban setting in Kenya: experiences and perceptions of women and healthcare providers | Kenya | Interpersonal; Organizational | Providers feel unsupported wtihin professional hierarchies; Stigma and discrimination | Qualitative |
| (Orpin, J.; Puthussery, S.; Burden, B.) | Healthcare providers’ perspectives of disrespect and abuse in maternity care facilities in Nigeria: a qualitative study | Nigeria | Interpersonal; Community | Stigma and discrimination; Perceptions of fitness for motherhood | Qualitative |
| (Patel, P.; Das, M.; Das, U.) | The perceptions, health-seeking behaviours and access of Scheduled Caste women to maternal health services in Bihar, India | India | Interpersonal; Community | Normalization of MoW; Poor regulation of private pecuniary interests; Pressure to achieve quantifiable public health goals; Stigma and discrimination | Qualitative |
| (Perera, D.; Lund, R.; Swahnberg, K.; Schei, B.; Infanti, J.J.; Darj, E.; Lukasse, M.; Bjørngaard, J.H.; Joshi, S.K.; Rishal, P.; Koju, R.; Pun, K.D.; Wijewardena, K.; Muzrif, M.M.; Campbell, J.C.; on behalf of the ADVANCE study team) | ‘When helpers hurt’: women’s and midwives’ stories of obstetric violence in state health institutions, Colombo district, Sri Lanka | Sri Lanka | Interpersonal; Organizational; Community | Internalized submission; Normalization of MoW; Perceptions around fitness for motherhood; Stigma and discrimination | Qualitative |
| (Rahangdale, L.; Banandur, P.; Sreenivas, A.; Turan, J.; Washington, R.; Cohen, C.R.) | Stigma as experienced by women accessing prevention of parent to child transmission of HIV services in Karnataka, India | India | Interpersonal; Community | Stigma and Discrimination; Perceptions regarding fitness for motherhood | Qualitative |
| (Sadler, M.; Santos, M.J.D.S.; Ruiz-Berdún, D.; Rojas, G.L.; Skoko, E.; Gillen, P.; Clausen, J.A.) | Moving beyond disrespect and abuse: addressing the structural dimensions of obstetric violence | Multiple (commentary) | Interpersonal; Organizational; Community; Law and Policy | Lack of conscientization; Health systems that do not earn users' trust; Normalization of MoW; Non-enforcement of laws, lack of accountability; Stigma and discrimination; Social and professional location of the providers | Commentary |
| (Sando, D.; Kendall, T.; Lyatuu, G.; Ratcliffe, H.; McDonald, K.; Mwanyika-Sando, M.; Emil, F.; Chalamilla, G.; Langer, A.) | Disrespect and abuse during childbirth in Tanzania: are women living with HIV more vulnerable? | Tanzania | Interpersonal | Health systems that do not earn users' trust | Mixed methods |
| (Siraj, A.; Teka, W.; Hebo, H.) | Prevalence of disrespect and abuse during facility based child birth and associated factors, Jimma University Medical Center, Southwest Ethiopia | Ethiopia | Interpersonal | Lack of conscientization; Stigma and discrimination; Social and professional location of the providers signifies something about their behavior towards women | Quantitative |
| (Sochas, L.) | Women who break the rules: Social exclusion and inequities in pregnancy and childbirth experiences in Zambia | Zambia | Interpersonal | Health systems that do not earn users' trust; Normalization of MoW; Stigma and discrimination; Pressure to achieve quantifiable public health goals | Qualitative |
| (Solnes Miltenburg, A.; van Pelt, S.; Meguid, T.; Sundby, J.) | Disrespect and abuse in maternity care: individual consequences of structural violence | Tanzania | Interpersonal; Organizational; Community | Lack of conscientization; Health systems that do not earn users' trust; Providers feel unsupported in professional hierarchies; Normalization of MoW; Stigma and discrimination | Qualitative |
| (Sri, B.S.; Sarojini, N.; Khanna, Renu) | An investigation of maternal deaths following public protests in a tribal district of Madhya Pradesh, central India | India | Organizational; Law and policy | Providers feel unsupported within professional hierarchies; Health systems that do not earn users' trust; Normalization of MoW; Non-enforcement of laws, lack of accountability; Stigma and discrimination; Policies as drivers of stigma; Pressure to achieve quantifiable public health goals | Qualitative |
| (Tekle Bobo, F.; Kebebe Kasaye, H.; Etana, B.; Woldie, M.; Feyissa, T.R.) | Disrespect and abuse during childbirth in Western Ethiopia: Should women continue to tolerate? | Ethiopia | Interpersonal; Organizational | Lack of conscientization; Companionship during institutional births; Normalization of MoW; Stigma and discrimination | Quantitative |
| (Turan, J.M.; Miller, S.; Bukusi, E.A.; Sande, J.; Cohen, C.R.) | HIV/AIDS and maternity care in Kenya: how fears of stigma and discrimination affect uptake and provision of labor and delivery services | Kenya | Interpersonal; Organizational | Providers feel unsupported within professional hierarchies; Stigma and discrimination | Qualitative |
| (Turan, J.M.; Bukusi, E.A.; Cohen, C.R.; Sande, J.; Miller, S) | EFFECTS OF HIV/AIDS ON MATERNITY CARE PROVIDERS IN KENYA | Kenya | Interpersonal; Organizational | Providers feel unsupported within professional hierarchies; Stigma and discrimination | Qualitative |
| (Upreti, M.; Jacob, J.) | The Philippines’ new postabortion care policy | Philippines | Law and policy | Policies as drivers of stigma | Commentary |
| (Upreti, M.; Jacob, J.) | The Philippines rolls back advancements in the postabortion care policy | Philippines | Law and policy | Policies as drivers of stigma | Commentary |
| (Vieira, N.; Rasmussen, D.N.; Oliveira, I.; Gomes, A.; Aaby, P.; Wejse, C.; Sodemann, M.; Reynolds, L.; Unger, H.W.) | Awareness, attitudes and perceptions regarding HIV and PMTCT amongst pregnant women in Guinea-Bissau– a qualitative study | Guinea-Bisseau | Interpersonal; Community | Providers feel unsupported wtihin professional hierarchies; Stigma and discrimination | Qualitative |
| (Wallace, H.Julie; McDonald, S.; Belton, S.; Miranda, A.; da Costa, E.; Matos, L.dC.; Henderson, H.; Taft, A.) | The decision to seek care antenatally and during labour and birth – Who and what influences this in Timor-Leste? A qualitative project exploring the perceptions of Timorese women and men | Timor-Leste | Interpersonal | Health systems that do not earn users' trust; Communication issues | Qualitative |
| (Warren, C.E.; Njue, R.; Ndwiga, C., & Abuya, T.) | Manifestations and drivers of mistreatment of women during childbirth in Kenya: Implications for measurement and developing interventions. | Kenya | Interpersonal; Organizational | Providers feel unsupported within professional hierarchies; Normalization of MoW; Stigma and discrimination; Non-enforcement of laws, lack of accountability; Social and professional location of providers signifies something about their behavior toward women | Qualitative |
| (Wassihun, B.; Deribe, L.; Worede, N.; Gultie, T.) | Prevalence of disrespect and abuse of women during child birth and associated factors in Bahir Dar town, Ethiopia | Ethiopia | Interpersonal | Communication issues; Stigma and discrimination | Quantitative |
| (Wassihun, B.; Zeleke, S.) | Compassionate and respectful maternity care during facility based child birth and women’s intent to use maternity service in Bahir Dar, Ethiopia | Ethiopia | Interpersonal | Stigma and discrimination | Quantitative |
| (Watson, H.L.; Downe, S.) | Discrimination against childbearing Romani women in maternity care in Europe: a mixed-methods systematic review | multiple in Europe (review) | Interpersonal | Lack of conscientization; Stigma and discrimination | Systematic review |
| (Yamin, A.E.; Galli, B.; Valongueiro, S.) | Implementing international human rights recommendations to improve obstetric care in Brazil | Brazil | Interpersonal; Law and policy | Health systems that do not earn users' trust; Non-enforcement of laws, lack of accountability; Stigma and discrimination; Poor regulation of private sector | Commentary |
| (Yasmine, R.; Moughalian, C.) | Systemic violence against Syrian refugee women and the myth of effective intrapersonal interventions | Lebanon | Interpersonal; Community; Organiztional; Law and policy | Health systems that do not earn users' trust; Communication issues; Perceptions around fitness for motherhood; Geopolitical and ethnopolitical projects | Commentary |
| (Yevoo, L.L.; Agyepong, I.A.; Gerrits, T.; van Dijk, H.) | Mothers’ reproductive and medical history misinformation practices as strategies against healthcare providers’ domination and humiliation in maternal care decision-making interactions: an ethnographic study in Southern Ghana | Ghana | Interpersonal | Health systems that do not earn users' trust; Stigma and discrimination | Qualitative |
| (Zordo, S.D.) | The biomedicalisation of illegal abortion: the double life of misoprostol in Brazil | Brazil | Interpersonal | Stigma and discrimination | Qualitative |
|  |  |  |  |  |  |
